# Fasting pancreatic polypeptide predicts incident microvascular and macrovascular complications of type 2 diabetes: An observational study

**DOI:** 10.1002/dmrr.3829

**Published:** 2024-07-01

**Authors:** Amir H. Sam, Adam J. Buckley, Brian Y.H. Lam, Gavin A. Bewick, Paul R. Bech, Karim Meeran, Maha T. Barakat, Stephen R. Bloom, Giles S.H. Yeo, Nader G. Lessan, Kevin G. Murphy

**Affiliations:** 1 Division of Diabetes, Endocrinology and Metabolism, Section of Endocrinology and Investigative Medicine, Imperial College London, London, UK; 2 Research Department, Imperial College London Diabetes Centre, Abu Dhabi, United Arab Emirates; 3 Wellcome-MRC Institute of Metabolic Science Metabolic Research Laboratories, Cambridge, UK; 4 Department of Diabetes and Obesity, King’s College London, London, UK

**Keywords:** diabetes mellitus, diabetic retinopathy, microvascular disease, pancreatic polypeptide, visceral adiposity

## Abstract

**Aims:**

Pancreatic polypeptide (PP) is elevated in people with vascular risk factors such as type 2 diabetes or increased visceral fat. We investigated potential relationships between PP and microvascular and macrovascular complications of diabetes.

**Materials and Methods:**

*Animal study*: Subcutaneous PP infusion for 4 weeks in high fat diet mouse model. Retinal mRNA submitted for Ingenuity Pathway Analysis. *Human study*: fasting PP measured in 1478 participants and vascular complications recorded over median 5.5 (IQR 4.9–5.8) years follow-up.

**Results:**

*Animal study*: The retinal transcriptional response to PP was indicative of cellular stress and damage, and this footprint matched responses described in previously published studies of retinal disease. Of mechanistic importance the transcriptional landscape was consistent with upregulation of folliculin, a recently identified susceptibility gene for diabetic retinopathy. *Human study*: Adjusting for established risk factors, PP was associated with prevalent and incident clinically significant retinopathy (odds ratio (OR) 1.289 (1.107–1.501) *p* = 0.001; hazard ratio (HR) 1.259 (1.035–1.531) *p* = 0.0213), albuminuria (OR 1.277 (1.124–1.454), *p* = 0.0002; HR 1.608 (1.208–2.141) *p* = 0.0011), and macrovascular disease (OR 1.021 (1.006–1.037) *p* = 0.0068; HR 1.324 (1.089–1.61), *p* = 0.0049), in individuals with type 2 diabetes, and progression to diabetes in non-diabetic individuals (HR 1.402 (1.081–1.818), *p* = 0.0109).

**Conclusions:**

Elevated fasting PP is independently associated with vascular complications of diabetes and affects retinal pathways potentially influencing retinal neuronal survival. Our results suggest possible new roles for PP-fold peptides in the pathophysiology of diabetes complications and vascular risk stratification.

## 1 Introduction

Intensive glycaemic control alone does not prevent microvascular and macrovascular complications of type 2 diabetes mellitus.^1,2^ Existing risk algorithms which incorporate additional previously established factors have limited predictive accuracy.^3^ Consequently, there is a need to identify new markers which can refine personalised risk estimates.

Central obesity, defined in terms of both waist circumference and weight-to-height ratio, significantly increases the incidence of both microvascular and macrovascular complications of diabetes.^4^ Increased visceral adipose tissue (VAT) volume has been identified as a potential additional risk factor for microvascular and macrovascular disease^5,6^ and incident type 2 diabetes.^7^ Intrahepatocellular lipid concentration (IHCLC) is independently associated with metabolic complications of obesity,^8^ and people in whom visceral adiposity coexists with high IHCLC are at the highest risk.^9^

We have previously demonstrated that fasting levels of the peptide hormone pancreatic polypeptide (PP) correlate with VAT volume and IHCLC.^10^ Copy number variation in the NPY Y4R gene, which encodes the cognate PP receptor, has been linked to variation in body mass index and waist circumference.^11,12^ Fasting PP increases with age and is also elevated in people with type 2 diabetes.^13,14^ Fasting PP is therefore of interest as a potential common marker linking several conditions associated with increased vascular disease risk.

We hypothesised that PP may play a physiological role in the relationship between adverse body composition and vascular risk. We therefore examined the effect of continuous PP infusion on retinal gene expression in a high-fat diet (HFD) mouse model, and performed a longitudinal cohort study to examine associations between fasting PP and prevalent and incident vascular complications in people with type 2 diabetes.

## 2 Research Design and Methods

### 2.1 Animal study

#### 2.1.1 Study design

Briefly, PP was infused continuously by subcutaneous osmotic pumps in a HFD mouse model and used for its physiological relevance, reproduction of vascular risk factors, and lack of severe metabolic derangements.^15^ The endpoint was differential retinal mRNA expression profile between the groups. To allow for inter-individual variation and outlier elimination, a group size of 15 was chosen.

The study was performed at Imperial College London under the Project License for ‘Energy Homoeostasis and Metabolism’ PPL 70/8068 and conducted according to ARVO principles for use of animals in visual research. Male C57BL/6J mice aged 6 weeks (Supporting Information S1) were randomised 1:1 to PP + vehicle or vehicle alone (https://randomizer.org) delivered by subcutaneously implanted Alzet model 1004 pumps over 28 days. PP was infused at 200 nmol/kg/day to maintain a serum level of 200–300 pmol/L, approximately mean post-prandial PP in humans with type 2 diabetes (Supporting Information S1). Following pump implantation (Supporting Information S1), animals were fed the D12492 (Research Diets, New Jersey, USA) diet, providing 60% of calories from fat. The treatment (PP) group was fed ad libitum, while the control group (PF) was pairfed 1.2 times the PP group’s previous day median intake (Supporting Information S1).

#### 2.1.2 Sample collection and bioinformatics analysis

Animals were sacrificed at 28 days following pump implantation (Supporting Information S1). Serum PP was measured in duplicate using an in-house radioimmunoassay which detects both murine and human PP (Inter-assay variation 13.8% at 28.5 pmol/L, 14.0% at 105 pmol/L and 14% at 191.1 pmol/L, correlation with commercial MILLIPLEX® Multiplex Luminex assay (Merck Millipore) *r* = 0.98, *p* < 0.001, see also Supporting Information S1).^16^ Eyes were embedded in OCT compound and cryostat sectioned prior to Laser Capture Microdissection. Total RNA was extracted and transcriptomes were submitted for Ingenuity Pathway Analysis (IPA) (Supporting Information S1).

### 2.2 Human study

#### 2.2.1 Setting and study design

The study used an observational design. Participants were recruited between 16/12/2014 and 01/09/2016 at Imperial College London Diabetes Centre, Abu Dhabi (ICLDC) by convenience sampling during routine clinical follow-up episodes. ICLDC specialises in diabetes and general endocrinology outpatient care, and provides primary care, specialist cardiology, nephrology and ophthalmology services and occupational health screening. The inclusion criterion was age ≥18 years and the exclusion criterion was non-type 2 diabetes mellitus. Participants provided single fasting venous blood samples at enrolment following written informed consent.

#### 2.2.2 Sample size and power

A total of 14 covariates were chosen for inclusion in the logistic regression analysis for the primary endpoint. The prevalence of retinopathy in people with type 2 diabetes in the MENA region has been estimated as up to 33.8%.^17^ Based on the predicted number of primary endpoint events and allowing 10 events per covariate, the aim was to recruit a minimum of (10 × 14)/0.338 = 414 participants with type 2 diabetes.

#### 2.2.3 Study endpoints

The primary microvascular endpoint was diabetic retinopathy. ‘Background’ eye disease was defined as background diabetic retinopathy (R1M0). ‘Significant’ eye disease was defined as higher-grade retinopathy or maculopathy (*R* ≥ 2 or M1). Digital retinal images were graded according to the International Council of Ophthalmology classification (2014). Where a specialist Ophthalmologist opinion was available, this superseded the digital retinal image grade. The highest grade of disease in either eye was recorded. The renal endpoint was persistent microalbuminuria without alternative diagnosis, defined as urinary albumin: creatinine ratio (ACR) ≥ 3.0 mg/mmol on separate occasions 3 months apart. Albuminuria was defined as ACR ≥30.0 mg/mmol.

The macrovascular endpoint was a composite of coronary artery, cerebrovascular, or peripheral arterial disease. Coronary artery disease was defined as myocardial infarction or acute coronary syndrome, regional wall motion abnormality on echocardiography, flowlimiting coronary artery stenosis, or coronary revascularisation. Cerebrovascular disease was defined as clinically or radiologically confirmed stroke. Peripheral vascular disease, defined as documented flow-limiting stenosis of a peripheral vessel, documented ulceration due to arterial insufficiency, or peripheral revascularisation, was included since 61% of patients with diabetes and peripheral vascular disease have coexisting coronary artery or cerebrovascular disease.^18^

#### 2.2.4 Sample collection and analysis

Samples for fasting PP were collected in 8 mL EDTA tubes, centrifuged at 4°C within 20 min, and stored at −80°C. Samples were assayed for PP in duplicate using in-house radioimmunoassay, as previously described (Supporting Information S1).^16^ Electrolytes, lipid profile and serum glucose were measured with the Cobas® 8000 analyser (Roche, Switzerland). HbA1c was measured using the Variant II Turbo system (Bio-Rad). Glomerular filtration rate (eGFR) was estimated using the CKD-EPI calculator. HOMA2-IR was determined using the HOMA calculator for insulin (Radcliffe Department of Medicine, Oxford). Missing baseline values were substituted with those from a recent attendance (< 4 months) for renal function (*n* = 29), HbA1c (*n* = 7) or lipid profile (*n* = 9).

#### 2.2.5 Statistical analysis

Analysis was performed using R 4.0.4 (R Foundation for Statistical Computing). Prism 6.1 (GraphPad Software) was used for data reduction of radioimmunoassays. Data are presented as median (interquartile range) and association measures as odds ratio (OR) (95% confidence interval) or hazard ratio (HR) (95% confidence interval). Skewed variables (Kolgorov-Smirnoff test and examination of Q-Q plot) were log-transformed (log2 PP = logPP). Binomial logistic regression was used to adjust for established risk factors for each endpoint studied. Because of a relatively large number of unaffected individuals, the data did not fit the Poisson distribution for estimation of risk ratios and the results are therefore presented as odds ratios. Multinomial logistic regression was performed using the *nnet* package and Cox proportional-hazards and Kaplan-Meier analyses used the *survival* and *survminer* packages. Proportional hazard assumptions were confirmed by statistical testing and visual inspection of Schoenfeld residuals. Inspection of Martingale residuals confirmed the use of logPP as the appropriate functional form in Cox regression analyses. Changes in the performance of nested models with the addition of logPP to established risk factors were assessed using Uno’s c-statistic, log-likelihood test and continuous Net Reclassification Index (NRI). Variance inflation factors for each covariate in each model were less than two, consistent with no significant multicollinearity. No outlier elimination was performed in the human study. No adjustment for multiple comparisons was performed due to the exploratory nature of the studies. Statistical significance was assessed at the level of *p* < 0.05.

## 3 Results

### 3.1 Continuous PP infusion in HFD-fed mice

#### 3.1.1 Differential retinal gene expression

**Figure 1:**
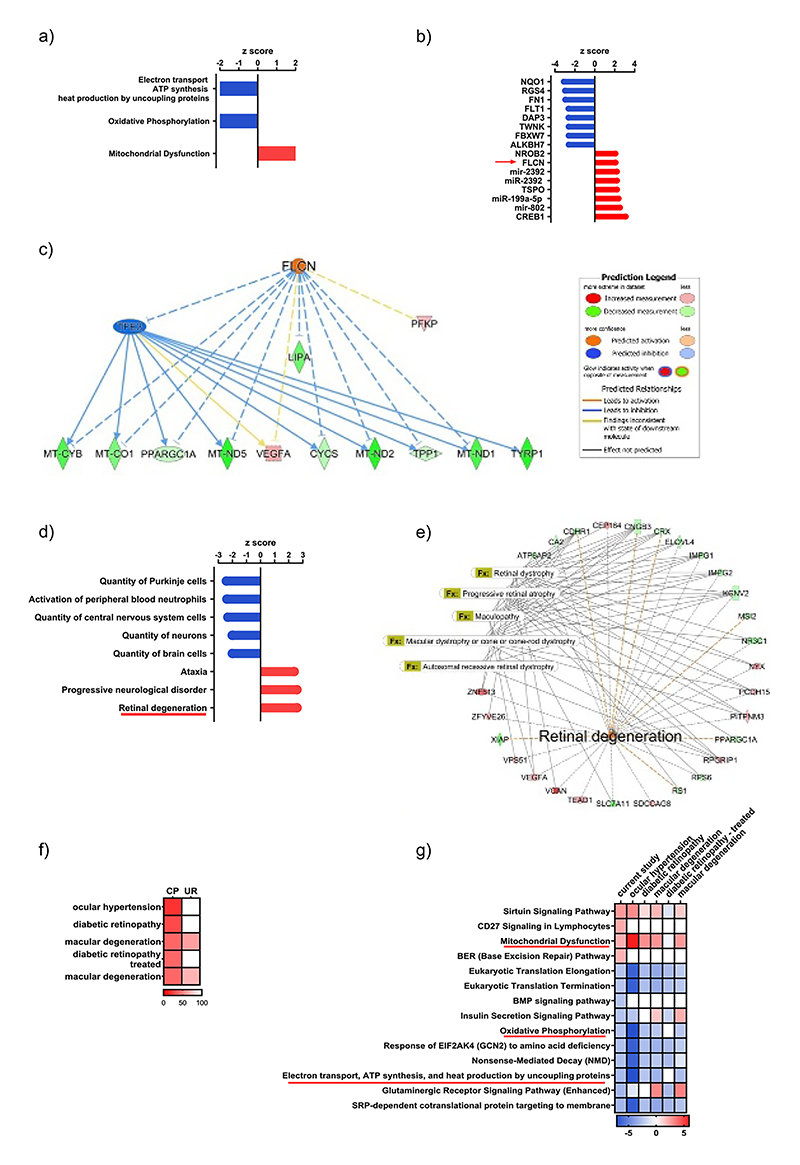
Ingenuity Pathway Analysis (IPA) of differential retinal gene expression between animals chronically treated with PP and controls (*n* = 10 in both groups following outlier elimination of 1st and 90th centiles). Panel (A) canonical pathways altered, (B) top eight upstream regulators up and down regulated, (C) Folliculin (FLCN) mechanistic network, (D) most and least enriched disease processes, (E) retinal degeneration-associated gene expression network with significantly enriched ophthalmic disease functions overlaid, (F) top matched human and mouse RNAseq analyses using IPA Analysis Match function, (G) comparison of effects on top canonical pathways between the present study and top matched analyses.

The dataset (*n* = 10 in each group following outlier elimination based on serum PP) was submitted for IPA (http://www.ingenuity.com/). A core analysis was conducted using a cutoff *p* value of <0.05 which reduced the analysis ready dataset to 868 genes, where 422 were upregulated and 446 were downregulated. Applying a stringent filter, <0.05 to the Benjamini-Hochberg multiple comparisons *p*-value and >2 for *z*-score, three canonical pathways were altered. Both the Electron transport chain ATP synthesis and heat production by uncoupling proteins (*z*-score −2.8, B-H -log *p*-value 1.319) and the Oxidative Phosphorylation (*z*-score −2.5, B-H -log *p*-value 1.319) pathways were down regulated, whereas the Mitochondrial dysfunction pathway (*z*-score 2.1, B-H -log *p*-value 1.319) was significantly activated (Figure 1A). This suggested defects in retinal cell energetics were evident and may be indicative of cellular stress or damage in the PP group. We next used IPA upstream regulator analysis to identify transcriptional regulators that might account for the observed gene changes in our dataset. We applied a *z*-score cutoff of >2 and a *p* value of overlap <0.05. In our dataset, Folliculin (FLCN), one of the top activated upstream regulators (Figure 1B), drives a mechanistic network predicted to inhibit TFE3 (Transcription Factor For Immunoglobulin Heavy-Chain Enhancer 3) and directly and indirectly through TFE3 influence 11 genes within our dataset (CYCS, LIPA, MT-CO1, MT-CYB, MT-ND1, MT-ND2, MT-ND5, PFKP, PPARGC1A, TPP1, TYRP1, VEGFA) many of which are associated with the Mitochondrial dysfunction pathway (Figure 1C). To predict the effects of our data set on downstream biological processes, we filtered the IPA Diseases and Functions analysis using a cut off *z*-score of >2. The most highly enriched disease process was retinal degeneration (*z*-score 2.5, *p* value 0.003), which contained 28 molecules from our dataset (Figure 1D). The retinal degeneration network is displayed in Figure 1E overlayed with selected significantly enriched ophthalmic disease functions. We next made use of IPA’s Analysis Match function, which automatically compared our analysis against over one hundred thousand other human, mouse, and rat expression analyses curated from public sources. We filtered the matches to include those for RNAseq of retina and mouse, excluded normal controls and then ordered by matched canonical pathway *z*-score, where a *z*-score over 50 is considered significant. The top matched analyses were all related to ophthalmic disease models and included mild ocular hypertension versus control (GSE122205, *z*-score 80.18), diabetic retinopathy induced by streptozotocin versus control (GSE87433, *z*-score 70.71), macular degeneration anti-ANGPT-TIE2-activating antibody versus mouse IgG antibody (GSE88730, *z*-score 59.76), diabetic retinopathy induced by strep-tozotocin with subsequent islet transplantation versus control (GSE87433, *z*-score 59.76) and macular degeneration VEGF-Trap versus mouse IgG antibody (GSE8873, *z*-score 59.76) (Figure 1F). Importantly, the significantly influenced canonical pathways in our dataset (Figure 1A) were similarly affected in the datasets highlighted by the match analysis except for the diabetic retinopathy model where mice had been treated for their diabetes by islet transplantation. For example, in this data set, the mitochondrial dysfunction pathway was not affected (Figure 1G). Taken together, the RNAseq data strongly suggest that chronic elevation of PP contributes to retinal damage and identifies possible mechanisms by which this may occur.

#### 3.1.2 PP did not influence growth, body composition or physiological parameters

At study termination, median (IQR) PP was 334 (290–446) pmol/L in the PP group and 53 (29–165) pmol/L in the PF group (*p* < 0.0001). Weight and Magnetic Resonance body composition immediately post-mortem did not differ significantly (Table S1). Fasted arterial blood glucose was elevated in both groups (10.5 ± 1.4 mmol/L PP, 10.5 ± 2.1 mmol/L PF, *p* = 0.99 between groups). Mean arterial blood pressure did not significantly differ between the groups.

### 3.2 Fasting PP as a predictor of vascular complications of diabetes in humans

#### 3.2.1 Study population and analysis

**Figure 2:**
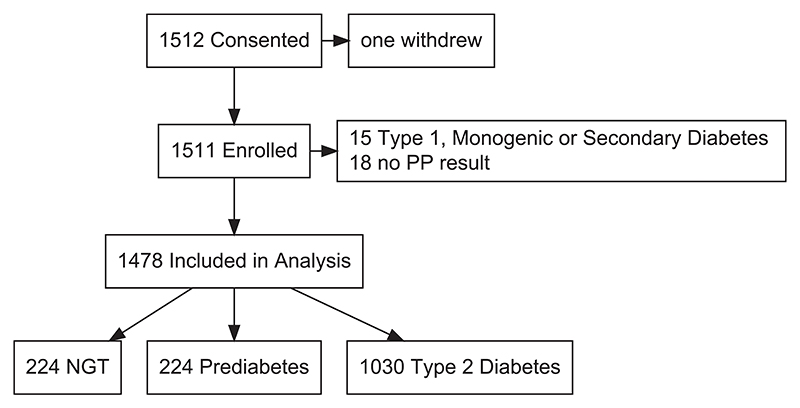
Flowchart illustrating the disposition of participants in the human study. Participants with an underlying diagnosis of Type 1, Monogenic, or Secondary Diabetes, or where the PP radioimmunoassay did not provide a result, were excluded from statistical analysis. NGT, normal glucose tolerance; PP, pancreatic polypeptide.

In total, 1512 individuals consented. One withdrew immediately following enrolment. Participants subsequently found to have Type 1, Monogenic, or secondary Diabetes Mellitus (*n* = 15) or without valid PP measurement due to assay failure (*n* = 18) were excluded (Figure 2). Baseline characteristics of 1478 included participants (224 NGT, 224 prediabetes, 1030 type 2 diabetes) are presented in Table 1. Participants were followed up over a median of 5.5 (4.9–5.8) years from recruitment. Fasting PP was weakly correlated with fasting insulin (*r* = 0.149, *p* < 0.0001), and eGFR (*r* = 0.068, *p* = 0.01) but not with BMI (*r* = 0.02, *p* = 0.52), in the study population as a whole. Fasting PP was weakly positively correlated with HOMA2-IR in individuals with type 2 diabetes not using exogenous insulin (*r* = 0.1, *p* < 0.05), but not significantly correlated with HOMA-%B or HOMA-%S (see also Table S2). Fasting PP was significantly increased in individuals using a sulfonylurea (26.0 (18.3–33.7) pmol/L, *p* < 0.001) or DPP4 inhibitor (12.2 (4.3–20.0) pmol/L, *p* < 0.01) at enrolment, adjusted for age, sex, use of other hypoglycaemic agents and diabetes status, consistent with their known effects on PP secretion^19^ and degradation^20^; however neither drug class was significantly associated with study endpoints, nor significantly interacted with the relationship between PP and study endpoints, and consequently both were excluded from further statistical analysis.

#### 3.2.2 Prevalence of diabetic retinopathy in people with type 2 diabetes

The relationship between logPP and prevalent diabetic retinal disease was examined in participants with type 2 diabetes, adjusted for the established risk factors of diabetes duration, systolic blood pressure (sBP), HbA1c, smoking and low-density lipoprotein (LDL) cholesterol in logistic regression. Because of missing retinal screening data in 8 cases, 1022 individuals were included (no retinopathy = 773, background = 78, significant = 171). LogPP was independently and significantly associated with prevalent significant retinopathy (OR 1.289 (1.107–1.501) *p* = 0.0011) but not background retinopathy (OR 1.211 (0.989–1.483) *p* = 0.0636), compared with individuals without retinal disease. Systolic blood pressure (sBP) was an independent predictor of significant retinopathy and HbA1c was a predictor of both background and significant retinopathy (Table 2). Addition of logPP significantly improved both model fit and continuous NRI for significant retinopathy (*x*^2^(1) = 9.24 *p* < 0.01, NRI (95% CI) = 0.25 (0.09–0.41) *p* < 0.01).

#### 3.2.3 Incidence of diabetic retinopathy in people with type 2 diabetes

**Figure 3:**
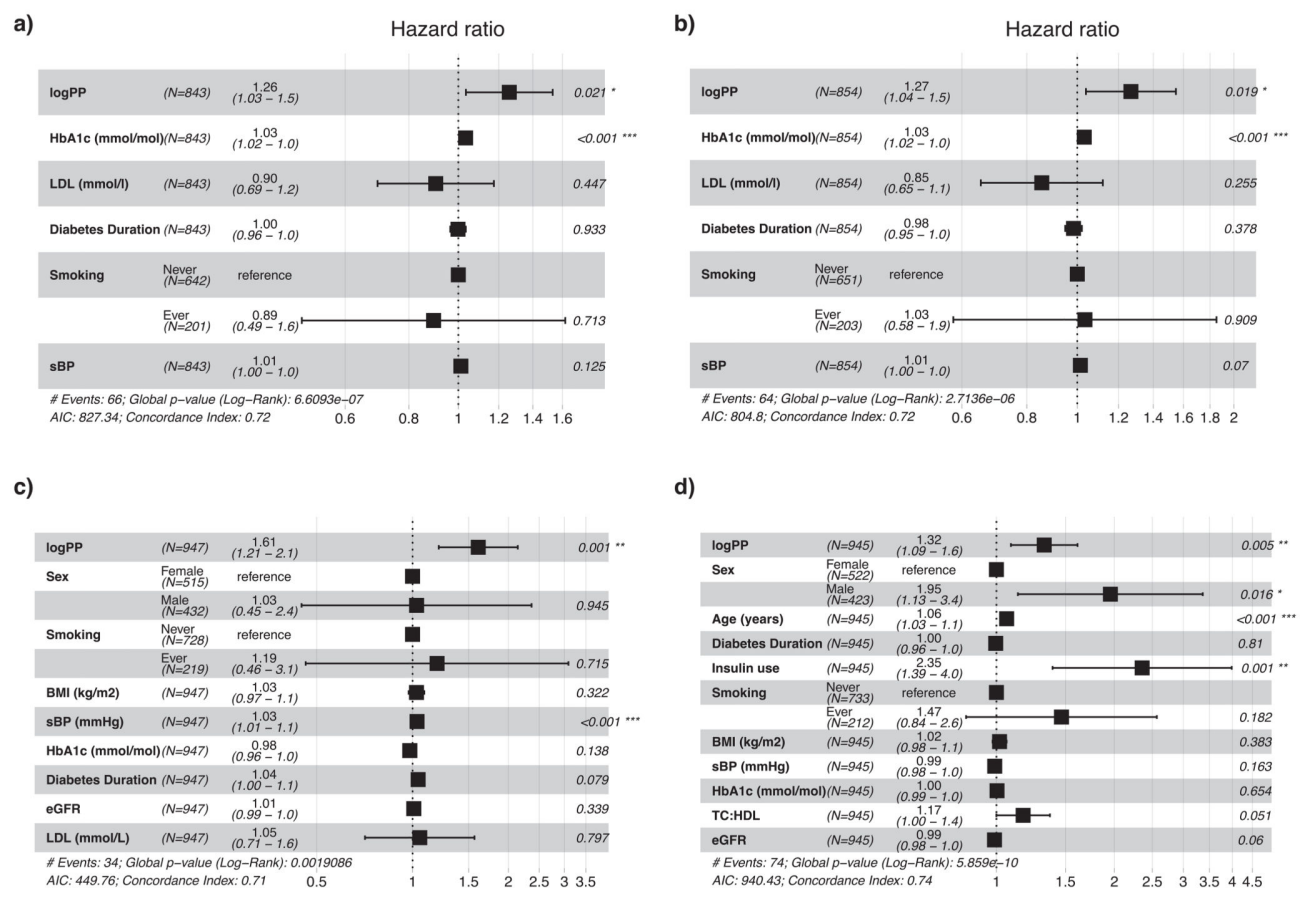
Summary of Cox regression analyses of logPP and established risk factors on incident vascular complications in participants with Type 2 Diabetes. Forest plots of logPP and established risk factors for prediction of (A) incidence of the composite significant retinal disease endpoint, (B) incident maculopathy, (C) incident nephropathy, (D) incident ASCVD. Diabetes Duration refers to the recorded duration of diabetes at the time of blood sample collection. eGFR, estimated glomerular filtration rate (ml/min/1.73m2); LDL, low-density lipoprotein; logPP, log2 fasting pancreatic polypeptide; sBP, systolic blood pressure; TC:HDL, ratio of total cholesterol to HDL-C.

Incident diabetic retinal disease was diagnosed through routine yearly clinical retinal screening over a median of 5.5 (4.8–5.8) years. Endpoints were onset of background retinopathy in individuals with no retinal disease (*n* = 745), incidence of macular oedema in individuals without maculopathy (*n* = 853), and incidence of the significant retinal disease endpoint in individuals without significant retinal disease at enrolment (*n* = 843). In univariate Cox proportional hazards analysis, logPP was significantly and positively associated with the composite microvascular endpoint (66 events, HR 1.386 (1.141–1.684, *p* < 0.001)). LogPP quartile was significantly associated with both incident significant retinal disease and incident maculopathy (HR 1.033 (1.021–1.045), *p* < 0.0001, 1.031 (1.019–1.044), *p* < 0.0001, log-rank test). Adjusting for HbA1c, LDL cholesterol, sBP, smoking, and diabetes duration, logPP was significantly associated with the composite endpoint (66 events, HR 1.259 (1.035–1.531), *p* = 0.0213) and incident maculopathy (64 events, HR 1.268 (1.039-1.548), *p* = 0.0195) but not incident background retinopathy (HR 1.006 (0.864–1.17), *p* = 0.9401). HbA1c was a significant predictor in each model (Figure 3A,B). Addition of logPP to the fully adjusted model resulted in modest improvement in prediction of significant retinopathy (Uno’s c-statistic (95% CI) 0.77 (0.68–0.86) with logPP included, 0.74 (0.62–0.85) without, increase in log-likelihood *χ*^2^ = 5.20 *p* < 0.05, continuous NRI 0.37).

#### 3.2.4 Prevalence of microalbuminuria in people with type 2 diabetes

Of 1030 individuals with type 2 diabetes, 291 (28.3%) had evidence of microalbuminuria at enrolment and 739 (71.7%) did not. Median fasting PP was 64.33 (36.36–95.61) pmol/L in individuals with microalbuminuria and 43.03 (24.38–83.67) pmol/L in individuals without. Adjusting for age, sex, BMI, diabetes duration, sBP, HbA1c, LDL cholesterol, eGFR, and smoking, logPP was an independent predictor of the nephropathy endpoint (OR 1.277 (1.124–1.454), *p* = 0.0002, Table 3). Examining the average marginal effect, a two-fold increase in logPP resulted in a 4.5% increase in the probability of the nephropathy endpoint. Addition of logPP improved model fit and continuous NRI (*x*^2^(1) = 14.17 *p* < 0.0001, NRI (95% CI) = 0.27 (0.14–0.41) *p* < 0.0001).

#### 3.2.5 Incidence of albuminuria in people with type 2 diabetes

Incidence of new onset or progression of renal disease during a median follow up period of 5.5 (4.9–5.8) years were defined as first recorded microalbuminuria (ACR of >3.0 mg/mmol in absence of preceding microalbuminuria, *n* = 710), or albuminuria (first ACR of >30.0 mg/mmol in absence of preceding albuminuria, *n* = 913). LogPP did not predict new onset of microalbuminuria (108 events, HR 1.06 (0.907–1.239), *p* = 0.4616) but did predict onset of albuminuria in unadjusted univariate (34 events, HR 1.483 (1.139–1.932), *p* = 0.003) and fully adjusted (34 events, HR 1.608 (1.208–2.141), *p* = 0.0011) analysis (Figure 3C). Fully adjusted model performance improved with inclusion of logPP (Uno’s c-statistic 0.70 (0.61–0.79) with, 0.65 (0.56–0.74) without, increase in log-likelihood *χ*^2^ = 8.97 *p* < 0.01, continuous NRI 0.26).

#### 3.2.6 Prevalence of the composite macrovascular endpoint in people with type 2 diabetes

Of all participants, 107 had at least one cardiovascular disease diagnosis at enrolment, an overall prevalence of 7.2%, compared with 100 (9.7%) in the type 2 diabetes group (Table 1). In the study population as a whole, median fasting PP was 73.8 (40.3–117.6) pmol/L in individuals meeting the cardiovascular endpoint and 36.3 (20.6–68.3) pmol/L in people who did not, as compared with 76.8 (48.5–123.4) pmol/L and 46.1 (26.7–84.1) pmol/L, respectively, in the type 2 diabetes group. Adjusting for age, sex, BMI, diabetes duration, HbA1c, insulin use, sBP, total cholesterol:HDL ratio (TC:HDL), statin use, smoking status and eGFR in the type 2 diabetes group alone, logPP was an independent predictor of the macrovascular endpoint (OR 1.021 (1.006–1.037), *p* = 0.0068, see Table 4). Examination of marginal effects indicated that a two-fold increase in fasting logPP increased the probability of being in the macrovascular disease group by 2.1%. With addition of logPP model fit and continuous NRI improved (*x*^2^(1) = 6.74 *p* < 0.01, NRI (95% CI) = 0.28 (0.07–0.50) *p* < 0.001).

#### 3.2.7 Incidence of the composite macrovascular endpoint in people with type 2 diabetes

The composite macrovascular endpoint was examined in 913 individuals with type 2 diabetes, and without recorded cardiovascular disease at enrolment. In univariate analysis, logPP was significantly associated with the macrovascular endpoint (74 events, HR 1.456 (1.216–1.744) *p* < 0.0001). Adjusted for sex, age, diabetes duration at enrolment, BMI, smoking, BMI, sBP, HbA1c, TC:HDL and eGFR (Figure 3D), logPP remained significantly and independently associated with the endpoint (74 events, HR 1.324 (1.089–1.610), *p* = 0.0049). Adjusted model performance improved slightly with addition of logPP (Uno’s c-statistic 0.74 (0.69–0.8) with, 0.72 (0.67– 0.78) without, increase in log-likelihood *χ*^2^ = 7.87 *p* < 0.01, continuous NRI 0.40).

#### 3.2.8 Association of fasting PP with progression to type 2 diabetes in people with euglycaemia or prediabetes

**Figure 4:**
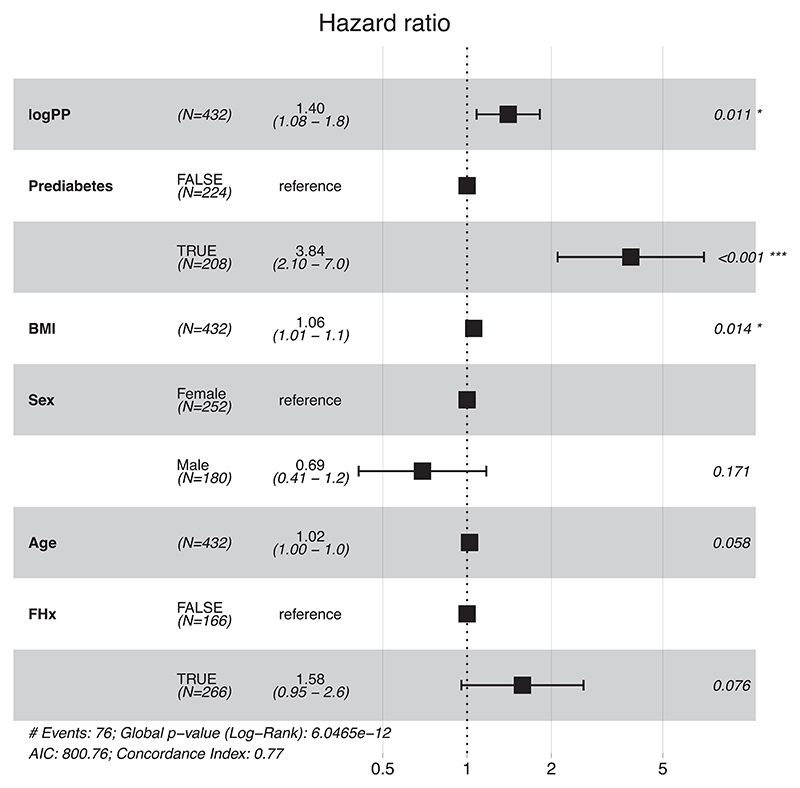
Cox proportional hazard regression of logPP, prediabetes, sex, age, and family history on progression to type 2 diabetes. A two-fold increase in PP is associated with a 1.402 (1.081–1.818), *p* = 0.0109 fold increase in the momentary risk of progression to type 2 diabetes in 432 individuals with normal glucose tolerance or prediabetes states at recruitment. Panels (A) forest plot of logPP and established risk factors for prediction of incident Type 2 Diabetes in individuals with normal glucose tolerance or prediabetes, (B) cumulative hazard of Type 2 Diabetes onset by quartile of fasting logPP. BMI, body mass index; FHx, positive family history of diabetes.

Progression to diabetes, defined as new clinical diagnosis and/or HbA1c of ≥6.5%, was assessed in 432 individuals with euglycaemia or prediabetes over a median follow-up period of 5.5 (3.2–6) years. In Cox regression, adjusted for glycaemic status at enrolment, BMI, sex, age at enrolment and family history of Diabetes, logPP was associated with increased HR for progression to diabetes (62 events in prediabetes group, 14 events in normal glucose tolerance group), HR 1.402 (1.081–1.818), *p* = 0.0109). Prediabetes diagnosis at enrolment and BMI were significantly associated with increased hazard of progression to diabetes (Figure 4). Addition of logPP increased model log-likelihood (*χ*^2^ = 6.37 *p* < 0.05 and continuous NRI (0.28).

## 4 Discussion

Our results demonstrate independent associations between fasting PP and microvascular and macrovascular risk in people with type 2 diabetes, despite comprehensive adjustment for established factors. In the human study, PP elevation preceded onset of both diabetes and diabetes complications. The performance of established risk factors in our study was comparable with previous reports.^3,21^ Addition of logPP improved model accuracy despite inclusion of important and unmodifiable risk factors such as age and diabetes duration, while logPP provided comparable or better accuracy in prediction of vascular complications than anthropometric methods.^22^

We found that fasting PP is not only elevated in individuals with existing type 2 diabetes^13,14^ but also increased in people who sub-sequently progress to type 2 diabetes, independent of age and BMI. This may reflect associations between PP and visceral adiposity,^10^ and visceral adiposity and vascular risk.^23,24^ Although PP secretion in response to food parallels insulin secretion, fasting PP was weakly correlated with fasting insulin and HOMA2-IR, and not correlated with HOMA-%B, suggesting that it is not simply a surrogate marker of insulin secretion or resistance. Vagal activity is the most important physiological stimulus for PP secretion^25^ and preservation of the vagus results in a reduction in loss of visceral fat following gastrectomy,^26^ suggesting that elevated vagal tone might contribute to the association between fasting PP and visceral adiposity.^10^ Conversely, intravenous administration of PP significantly reduces both efferent and afferent vagal nerve activity^27^ and inhibition of hepatic vagal tone impairs the compensatory reduction in glycogenolysis which occurs in response to infusion of long chain fatty acids^28^ resulting in net increased hepatic glucose production.^28^ Consequently, negative feedback effects of tonically elevated PP on the hypothalamus and vagal nuclei might, in turn, deleteriously affect glucose tolerance.

Associations between PP, central obesity and vascular disease may alternatively be attributable to a direct effect of PP signalling. Copy number variation analyses of the 10q11.22 locus, corresponding to the NPY Y4R gene, show associations with both obesity and waist circumference.^11,12^ NPY Y4R mRNA is expressed in the human brain and coronary artery.^29^ NPY Y4R is also expressed in multiple retinal cell types including photoreceptors, amacrine cells and gan-glion cells, as well as being diffusely distributed throughout the inner and outer plexiform layers.^30^ Finally, NPY receptor mRNA is expressed in human neutrophils, with NPY Y4R having the highest level of expression,^31^ suggesting a possible effect on systemic inflammatory pathways. It is consequently plausible that signalling at NPY receptors provides novel mechanisms linking adverse body composition with the incidence of vascular complications of diabetes.

The differential changes in retinal gene expression observed in the absence of measurable differences in blood glucose, body composition or blood pressure in the animal study suggest possible direct effects on neuronal survival. Interestingly, Folliculin (FLCN), one of the top activated upstream regulators, has been recently identified as a disease gene for diabetic retinopathy.^32^ FLCN has been identified as a negative regulator of *PPARGC1A*, a transcriptional coactivator which promotes mitochondrial biogenesis, and increases oxidative phosphorylation^33^ as well as protecting against reactive oxygen species^34^ and endoplasmic reticulum stress.^35^ FLCN has also been shown to suppress 5′AMP-activated protein kinase (AMPK), an important regulator of cellular energy metabolism which promotes autophagy and confers resistance to oxidative stress.^36^ Inhibition of autophagy sensitises the retina to light-induced retinal cell death,^37^ promotes photoreceptor cell death,^38^ and results in activation of the inflammasome and promotion of angiogenesis as well as loss of photoreceptors.^39^ Autophagy protects retinal pigment epithelium cells and retinal neuronal cells exposed to high external glucose concentrations from apoptosis.^40^ Consequently, the upregulation of *FLCN* in response to tonic elevation of PP in the mouse model suggests a potential causative relationship between elevated fasting PP and the risk of diabetic retinopathy.

### 4.1 Potential sources of bias and limitations

People with more severe or complicated diabetes may be reviewed more frequently and therefore over-represented. The retinopathy endpoint relies on multiple different clinical sources without systematic standardisation. Mortality data was unavailable, which may have resulted in under-reporting of endpoints during follow-up. With respect to the animal study, the lifespan of the osmotic pumps necessitated a duration of 4 weeks, which was too brief a period to assess changes in vascular histology, and measuring protein expression in addition to gene expression would further support the conclusions of the IPA. Although there is evidence that hyperglycaemia was induced in both groups of HFD-fed mice, the overall glucose burden may have been relatively mild. We did not know in advance how successful we would be in recovering retinal RNA or which retinal components might be most affected, and consequently sampled the full thickness of the retina rather than specific call populations.

## 5 Conclusions

Elevation of fasting PP is independently associated with, and precedes, both prevalent and incident microvascular and macrovascular disease in individuals with type 2 diabetes, whereas in individuals with normoglycaemia or prediabetes, fasting PP is associated with increased incidence of diabetes onset. Tonic elevation of PP in a mouse model results in upregulation of susceptibility genes for diabetic retinopathy. Further work is required to investigate the utility of PP as a biomarker for diabetes-associated vascular disease.

## Supplementary Material

Supporting Information

## Figures and Tables

**Table 1:** Characteristics of 1478 participants included in statistical analysis.

	NGT (*n* = 224)	Pre (*n* = 224)	T2DM (*n* = 1030)
Female	145 (65%)	119 (53%)	557 (54%)
Fasting PP (pmol/L)	18.7 (13.3–31.5)	26.3 (16.3–43.9)	49.1 (27.4–88.6)
Age (years)	41.1 (31.0–49.9)	48.9 (39.2–56.8)	55.9 (49.1–62.5)
Arab Emirati	213 (95%)	204 (91%)	910 (88%)
BMI (kg/m^2^)	28 (24.5–32.3)	31.5 (27.9–34.7)	31.4 (27.8–35.6)
sBP (mmHg)	116 (107–126.2)	125 (114–136)	128.5 (117–140)
dBP (mmHg)	71 (64–78)	74 (66–81)	73 (66–80)
Duration T2DM or prediabetes (years)	0	4.8 (0.9–10.8)	5 (0.8–11)
HbA1c (mmol/mol)	34 (31–36)	38 (35–41)	54 (46–64)
TC (mmol/L)	4.75 (4.14–5.37)	4.67 (3.95–5.34)	4 (3.45–4.7)
HDL (mmol/L)	1.44 (1.23–1.67)	1.29 (1.1–1.51)	1.18 (0.98–1.41)
TC:HDL	3.25 (2.77–3.95)	3.57 (2.86–4.42)	3.34 (2.78–4.16)
TG (mmol/L)	0.96 (0.71–1.31)	1.13 (0.88–1.58)	1.34 (1–1.83)
eGFR (mL/min/1.73m^2^)	98.4 (86.9–108.5)	101.1 (88.6–109.2)	99.6 (88.3–109)
Insulin (pmol/L)	74.0 (48.5–98.5)	102.6 (70.4–144.9)	102.4 (68.8–148.1)
HOMA2-IR	1.4 (0.9–1.9)	1.9 (1.3–2.8)	2.1 (1.4–3.2)
Statin treatment	41 (18%)	108 (48%)	883 (86%)
Number antihypertensives	0 (0–0)	0 (0–0)	0 (0–1)
Number oral hypoglycaemic agents	0 (0–0)	0 (0–0)	2 (1–3)
Insulin treatment	0 (0%)	0 (0%)	237 (23%)
Retinal disease (nil/BG/Sig/NA)	34/0/0/190 (15%/0%/0%/85%)	90/1/0/133 (40%/0%/0%/60%)	773/78/171/8 (75%/7%/17%/1%)
Macrovascular disease (CAD/CVD/PVD/total)	1/1/0/2 (0.5%/0.5%/0%/1%)	3/2/0/5 (1.3%/1%/0%/2.3%)	81/11/13/100 (8%/1%/1%/10%)

*Note*: Values are expressed as median (interquartile range).

Abbreviations: BG, background retinal disease; CAD, coronary artery disease; CVD, cerebrovascular disease; dBP, diastolic blood pressure; eGFR, estimated glomerular filtration rate (CKD-EPI); HDL, high density lipoprotein; NA, data not available; NGT, normal glucose tolerance; PP, pancreatic polypeptide; Pre, impaired fasting glucose or impaired glucose tolerance; PVD, peripheral vascular disease; sBP, systolic blood pressure; Sig, significant retinal disease; T2DM, type 2 diabetes mellitus; TC, total cholesterol; TG, triglycerides.

**Table 2:** Multinomial logistic regression of logPP and established risk factors on prevalent retinopathy endpoints at recruitment in participants with type 2 diabetes.

Group	Coefficient	OR (95% CI)	*p*
Background	logPP	1.211 (0.989–1.483)	= 0.0636
	HbA1c (mmol/mol)	1.032 (1.018–1.046)	<0.0001***
	LDL (mmol/L)	0.956 (0.740–1.235)	= 0.7308
	Diabetes duration	1.005 (0.972–1.038)	= 0.7740
	Ever smoked	0.803 (0.449–1.437)	= 0.4599
	sBP (mmHg)	1.007 (0.993–1.021)	= 0.3521
Significant	logPP	1.289 (1.107–1.501)	= 0.0011**
	HbA1c (mmol/mol)	1.045 (1.035–1.056)	<0.0001***
	LDL (mmol/L)	0.776 (0.634–0.950)	= 0.0139*
	Diabetes duration	1.000 (0.975–1.025)	= 0.9733
	Ever smoked	0.808 (0.523–1.248)	= 0.3373
	sBP (mmHg)	1.023 (1.013–1.034)	<0.0001***

Abbreviations: B, standardised coefficient; LDL, low-density lipoprotein; LogPP, log2 fasting pancreatic polypeptide; OR, odds ratio (95% confidence interval); *p* = significance; sBP, systolic blood pressure; SE, standard error.

**Table 3:** PP is a significant predictor of the nephropathy endpoint in logistic regression.

Coefficient	OR (95% CI)	*p*
logPP	1.277 (1.124–1.454)	=0.0002***
Age (years)	1.016 (1.000–1.032)	=0.0448*
Male sex	1.438 (1.008–2.049)	=0.0442*
BMI (kg/m^2^)	1.029 (1.003–1.055)	=0.0258*
Diabetes duration	1.003 (0.981–1.025)	=0.7749
sBP (mmHg)	1.025 (1.016–1.034)	<0.0001***
HbA1c (mmol/mol)	1.018 (1.009–1.027)	=0.0001***
LDL (mmol/L)	0.905 (0.764–1.068)	=0.2408
eGFR (ml/min/1.73m2)	1.002 (0.993–1.010)	=0.7189
Ever smoked	0.965 (0.644–1.442)	=0.8616

Abbreviations: B, standardised coefficient; BMI, body mass index; eGFR, estimated glomerular filtration rate; LDL, low-density lipoprotein; logPP, log2 fasting PP; OR, Odds ratio (95% confidence interval); *p* = significance; sBP, systolic blood pressure; SE, standard error.

**Table 4:** PP is a significant predictor of the macrovascular endpoint in individuals with type 2 diabetes.

Coefficient	OR (95% CI)	*p*
logPP	1.021 (1.006–1.037)	= 0.0068**
Age (years)	1.006 (1.004–1.008)	<0.0001***
Male sex	1.081 (1.036–1.128)	= 0.0003***
BMI (kg/m^2^)	1.000 (0.997–1.003)	= 0.7972
Diabetes duration	1.001 (0.998–1.003)	= 0.5521
HbA1c (mmol/mol)	1.000 (0.999–1.001)	= 0.9418
TC:HDL	1.012 (0.998–1.026)	= 0.0897
Insulin use	1.070 (1.023–1.120)	= 0.0033**
sBP (mmHg)	0.999 (0.998–1.000)	= 0.0448*
Statin use	0.970 (0.922–1.022)	= 0.2527
Ever smoked	1.022 (0.973–1.073)	= 0.3808
eGFR (ml/min/1.73m^2^)	0.999 (0.998–1.000)	= 0.1094

*Note*: Logistic regression of predictors on the macrovascular disease endpoint in participants with type 2 diabetes mellitus. Nagelkerke *R*^2^ = 0.302.

Abbreviations: B, standardised coefficient; BMI, body mass index; eGFR, estimated glomerular filtration rate; LogPP, log2 fasting PP; OHA, oral hypoglycaemic agent; OR, odds ratio (95% confidence interval); *p* = significance; sBP, systolic blood pressure; SE, standard error; TC: HDL, ratio of total cholesterol to HDL.

## Data Availability

Data that underlie the results reported in this article after de-identification (text, tables, and figures) will be available. Data will be available for up to 5 years following publication for sharing with researchers who submit a study question which, in the opinion of the authors, can reasonably be addressed by the data. Enquiries should be directed to abuckley@icldc.ae. An institutional contract and data sharing agreement will be required.
