# Supplementary material for: Fasting pancreatic polypeptide predicts incident microvascular and macrovascular complications of type 2 diabetes: An observational study
Source: Diabetes Metab Res Rev. Author manuscript; Available in PMC 2025 Mar 14. (PMC7617488; doi:10.1002/dmrr.3829)
Supplement: Supporting Information [file EMS203506-supplement-Supporting_Information.docx]

# Supplementary Methods

## 1.0 Animal study

*1.1 Study animals and animal welfare*

Male C57BL/6J mice were obtained from Charles River (Margate, UK) at age 6 weeks. Study animals were acclimated for 7 days at a standard temperature of 22˚C and at 55% humidity, with a standard 12 hour light / 12 hour dark diurnal cycle for seven days, with water ad libitum and standard mouse chow (RM1 diet, Special Diets Services, UK), in order to assess fitness for surgery. Animals were handled daily during this period in order to familiarise them with the study operator and equipment, using the cupped hand and tunnel techniques in order to minimise interference with physiological parameters due to stress. Handling was performed by a single investigator to control for inter-operator effects.

*1.2 Study agent and dosing*

In healthy human adults in their fifth decade, fasting PP is 30 ± 23 pmol/L and postprandial PP is 175 — 160 pmol/L ^1^. In humans, subcutaneous infusion of bovine PP at 2 pmol/kg/min (equivalent to 2.88 nmol/kg/day) increased plasma PP to between 450 and 700 pmol/L ^2^. In the mouse, infusion of PP via Alzet pump model 1003 at 250 nmol/kg/day for four days resulted in plasma PP of 300-400 pmol/L ^3^. An infusion rate of 200 nmol/kg/day was therefore chosen to maintain a target serum PP of 200-300 pmol/L. Recombinant human PP was used as the study agent since it is known to bind the rodent Y4 receptor with high affinity ^4^. The Alzet model 1004 pump was chosen since it is rated to infuse for a minimum of four weeks and since re-implantation of pumps was considered to be an excessive level of severity for an exploratory study. Recombinant human PP was reconstituted in sterile water (5% by volume), 0.1M acetic acid (18%) and saline (77%).  Alzet model 1004 osmotic pumps were loaded with 100 µl of PP+vehicle or vehicle alone after filtration using Millex GP 0.22 µm anti-bacterial filters, and primed for 48 hours in 0.9% saline maintained at 37˚C.

*1.3 Pump implantation*

Following acclimatisation, study animals were randomised 1:1 to PP+vehicle or vehicle alone ([https://randomizer.org](https://randomizer.org/)).  Pumps were implanted in the interscapular region under isoflurane anaesthesia.  Induction was performed with an oxygen flow rate of 1.5 L/min and anaesthetic entrained at 5%. Animals were transferred to a Bain coaxial circuit with a scavenger unit, using an oxygen flow rate of 0.8 L/min and isoflurane entrained at 2% for maintenance of anaesthesia. A warming pad set to 30˚C was used during surgery. Sufficient depth of anaesthesia was assessed by means of the pedal withdrawal reflex. Wound closure was achieved with surgical clips, removed after five days. Buprenorphine 0.1 mg/kg sc was administered prior to recovery from anaesthesia. Mice were kept in a recovery pen at 35˚C until conscious, and thereafter returned to individual cages. All animals recovered fully following surgery with no symptoms or behaviours suggestive of uncontrolled pain or infection.

*1.4 Physiological monitoring and pair feeding*

All study animals were weighed daily. Intake was assessed by weighing each animal’s chow daily at the beginning of the light phase. The treatment (PP) group was fed ad libitum, the control group (PF) was pair-fed 1.2 times the PP group previous day median intake, accounting for PP effects on satiety. The order in which animals were weighed and fed was randomised for each day of the study. During the final week, mean arterial pressure was measured using the CODA monitor system (Kent Scientific Corporation, Torrington, USA).

*1.5 Sample collection*

Animals were humanely killed under Schedule 1 by cervical dislocation without sedation followed by decapitation, at 28 days after pump insertion. Blood glucose was measured using a portable monitor system. Blood was collected from the carotid arteries into serum tubes, centrifuged, and serum was stored at -80˚C. Inguinal and epididymal fat pads were weighed individually. Tissue samples were flash frozen in liquid nitrogen immediately and stored at -80˚C. Osmotic pumps were excised and stored. Post-mortem MRI body composition analysis was performed using the EchoMRI-100H system (EchoMRI, Houston, TX).

## 2.0 Pancreatic Polypeptide Assay

Samples were assayed in duplicate using in-house radioimmunoassay, as previously described ^5^. Inter-assay variation was 13.8% at 28.5 pmol/L, 14.0% at 105 pmol/L and 14% at 191.1 pmol/L. This assay has excellent consistency (Pearson correlation coefficient 0.98, p < 0.001) with the commercial MILLIPLEX® Multiplex Luminex assay (Merck Millipore, Billerica, MA, USA) ^6^. Fasting PP, repeated in 30 cases, was well correlated between visits in human participants (Spearman test, rho=0.771, p<0.0001) indicating good reproducibility. For murine samples a five-fold dilution was used to fit the standard curve and consequently the lower limit of detection was 25 pmol/L.

Gut hormone radioimmunoassays use polyclonal rabbit antibodies, and hence interference from human anti-rabbit antibodies in people who keep rabbits can result in significant false elevate of apparent levels of PP two orders of magnitude greater than the actual value ^7^. Patients who have been treated with United States Pharmacopeia (USP) insulin, which was derived from beef and pork pancreas and contained a high molecular weight form of pancreatic polypeptide, develop antibodies to PP which can interfere with measurement ^8^. Pet ownership is limited in the United Arab Emirates, and patients attending ICLDC almost exclusively use modern insulin analogues and consequently the study results are unlikely to be subject to significant assay interference.

**3.0 mRNA extraction and analysis**

Ten µm retinal sections were dehydrated using sequential ethanol washes before Laser Capture Microdissection using the Zeiss PALM MicroBeam system as previously described ^9^.  Total RNA was extracted using RNeasy Plus Micro kits (Qiagen, Netherlands).  RNA quality was assessed using Agilent Bioanalyzer 2100 (Agilent, USA) with a RIN cut-off of 6.  Sequencing libraries were generated using SMARTer Stranded Total RNA-seq – Pico Input kit (V2) (Takara Bio, Japan), and paired-end 50bp sequencing performed using the Illumina NovaSeq 6000 instrument (Illumina, USA).  Following outlier elimination of < 10th and > 90th centiles of serum PP, treatment and paired-fed transcriptomes were compared using Ingenuity Pathway Analysis version 49309495 (Qiagen). Reads were mapped to mouse GRCm38 genome and genes quantitated using STAR (2.5.0a) using default parameters.  Differential gene expression was determined using gene-wise likelihood ratio tests from EdgeR (3.34.0)/R 4.1.0, with significance threshold set at uncorrected p<0.05.

**Supplementary Table 1: Body composition of 30 mice treated with high fat diet (HFD) + PP or HFD + vehicle for four weeks**. Results are presented as mean ± sd. P-values represent differences between groups by two-tailed Student’s t-test. EAT = Epididymal fat pad weight, IAT = inguinal fat pad weight, EAT:IAT = ratio of weight of epididymal fat pad to inguinal fat pad weight.

| **Variable** | **HFD + PP** (n=15) | **HFD + Vehicle** (n=15) | **P value** |
| --- | --- | --- | --- |
| Fat mass (g) | 7.75 ± 2.42 | 6.91 ± 2.24 | 0.33 |
| Lean mass (g) | 19.21 ± 1.35 | 18.98 ± 1.31 | 0.64 |
| Free water | 0.07 ± 0.04 | 0.09 ± 0.04 | 0.22 |
| Total water | 15.55 ± 1.32 | 15.24 ± 0.88 | 0.46 |
| EAT (g) | 1.15 ± 0.46 | 0.99 ± 0.37 | 0.30 |
| IAT (g) | 0.82 ± 0.33 | 0.71 ± 0.27 | 0.34 |
| EAT:IAT | 1.39 ± 0.15 | 1.39 ± 0.15 | 0.92 |
| Fat:Lean | 0.4 ± 0.11 | 0.36 ± 0.1 | 0.29 |

**Supplementary Table 2: Univariate correlations between baseline characteristics and fasting plasma Pancreatic Polypeptide**

|  | **NGT** | **Pre** | **T2DM** | **All** |
| --- | --- | --- | --- | --- |
| BMI (kg/m2) | r(222)=0.128, p=0.06. | r(222)=-0.133, p<0.05 * | r(1028)=-0.094, p<0.01 ** | r(1476)=0.007, p=0.78 |
| Weight (kg) | r(222)=0.21, p<0.01 ** | r(222)=-0.089, p=0.17 | r(1028)=-0.08, p<0.01 ** | r(1476)=0.017, p=0.52 |
| Age (years) | r(222)=0.359, p<0.0001 *** | r(222)=0.295, p<0.0001 *** | r(1028)=0.239, p<0.0001 *** | r(1476)=0.395, p<0.0001 *** |
| HbA1c (%) | r(222)=0.197, p<0.01 ** | r(222)=0.243, p<0.01 *** | r(1028)=0.168, p<0.0001 *** | r(1476)=0.404, p<0.0001 *** |
| HOMA2IR | r(222)=0.118, p=0.08 . | r(222)=-0.038, p=0.58 | r(1028)=0.011, p=0.72 | r(1476)=0.119, p<0.0001 *** |
| HOMA2B | r(222)=0.117, p=0.09. | r(222)=-0.067, p=0.33 | r(1028)=-0.073, p<0.05 * | r(1476)=-0.211, p<0.0001 *** |
| HOMA2S | r(222)=-0.116, p=0.088 . | r(222)=0.036, p=0.60 | r(1028)=-0.01, p=0.74 | r(1476)=-0.118, p<0.0001 *** |
| Insulin (pmol/L) | r(222)=0.119, p=0.079 . | r(222)=-0.023, p=0.73 | r(1028)=0.006, p=0.84 | r(1476)=0.085, p<0.01 ** |
| eGFR (ml/min/1.73m2) | r(222)=0.152, p<0.05 * | r(222)=-0.026, p=0.71 | r(1028)=0.059, p=0.061 . | r(1476)=0.061, p<0.05 * |

## References

1. Valenzuela JE, Taylor IL, Walsh JH. Pancreatic polypeptide response in patients with chronic pancreatitis. *Digestive diseases and sciences*. 1979; 24: 862-864.

2. Rabiee A, Galiatsatos P, Salas-Carrillo R, Thompson MJ, Andersen DK, Elahi D. Pancreatic polypeptide administration enhances insulin sensitivity and reduces the insulin requirement of patients on insulin pump therapy. *Journal of diabetes science and technology*. 2011; 5: 1521-1528.

3. Sam AH, Gunner DJ, King A et al. Selective ablation of peptide YY cells in adult mice reveals their role in beta cell survival. *Gastroenterology*. 2012; 143: 459-468.

4. Tough IR, Holliday ND, Cox HM. Y(4) receptors mediate the inhibitory responses of pancreatic polypeptide in human and mouse colon mucosa. *The Journal of pharmacology and experimental therapeutics*. 2006; 319: 20-30.

5. Adrian TE, Bloom SR, Bryant MG, Polak JM, Heitz PH. Proceedings: Radioimmunoassay of a new gut hormone-human pancreatic polypeptide. *Gut*. 1976; 17: 393-394.

6. Sam AH, Sleeth ML, Thomas EL et al. Circulating pancreatic polypeptide concentrations predict visceral and liver fat content. *The Journal of clinical endocrinology and metabolism*. 2015; 100: 1048-1052.

7. Ramachandran R, Bech P, Dhillo W et al. Rabbit interference with gut peptide immunoassay. *Regulatory peptides*. 2010; 164: 15-16.

8. Bauman WA, Meryn S. A high molecular weight form of pancreatic polypeptide was present in USP insulin and is absent in a more recent insulin preparation. *Journal of Pharmacy and Pharmacology*. 1987; 39: 549-550.

9. Polex-Wolf J, Lam BY, Larder R et al. Hypothalamic loss of Snord116 recapitulates the hyperphagia of Prader-Willi syndrome. *J Clin Invest*. 2018; 128: 960-969.
